# Incidence and progression of osteoarthritis following curettage and cementation of giant cell tumor of bone around the knee: long-term follow-up

**DOI:** 10.1186/s10195-023-00693-8

**Published:** 2023-04-06

**Authors:** Walid Atef Ebeid, Ismail Tawfeek Badr, Mohamed Kamal Mesregah, Bahaa Zakarya Hasan

**Affiliations:** 1 Department of Orthopaedic Surgery, Cairo University, Cairo, Egypt; 2 Department of Orthopaedic Surgery, Menoufia University, Shebin El-Kom, Menoufia Egypt

**Keywords:** Giant cell tumor, Knee, Curettage, Bone cement, Osteoarthritis, Kellgren–Lawrence grading

## Abstract

**Background:**

Giant cell tumor of bone (GCTB) is a benign locally aggressive tumor frequently treated with intralesional curettage and cementation. The aim of this study was to investigate the long-term incidence of arthritic changes following curettage and cementation of GCTB around the knee.

**Materials and methods:**

This study was a retrospective review of patients with GCTB around the knee treated with curettage and cementation with a minimum follow-up of 10 years. The functional results were assessed using the Musculoskeletal Tumor Society (MSTS) score. The arthritic changes were classified using the Kellgren–Lawrence (KL) classification system of osteoarthritis.

**Results:**

This study included 119 patients, 54 males and 65 females, with a mean age of 29.4 ± 9.2 years. There were 35 (29.4%) patients with pathological fractures. There were 84 (70.6%) patients with de novo lesions and 35 (29.4%) with recurrent lesions. The mean follow-up period was 13.2 ± 3.16 years. The mean MSTS score was 28.5 ± 1.9. Overall, 25 (21%) patients developed variable degrees of arthritis of KL grade 1 (*n* = 7), KL grade 2 (*n* = 11), KL grade 3 (*n* = 4), and KL grade 4 (*n* = 3). Ten patients showed progression of arthritis during the follow-up period. Age at presentation, gender, presence of pathological fracture, whether the tumor was de novo or recurrent, and tumor location were not associated with arthritis incidence.

**Conclusions:**

Curettage and cementation can be used safely to treat GCTB around the knee. Arthritis of the knee is a possible complication, but mild grades are expected in most cases. There was no association between arthritis incidence and age, gender, pathological fractures, tumor location, or recurrent tumors.

**Level of evidence:**

Level IV.

## Introduction

Giant cell tumor of bone (GCTB) is a relatively rare locally aggressive benign primary bone tumor. Approximately half of these tumors occur around the knee and usually invade the subchondral bone. These tumors rarely metastasize to the lung (1–4%) and may present with a pathological fracture [1–4]. GCTB usually occurs in young adults in the 2nd to 4th decades of life [2, 5].

A high incidence of recurrence following surgical treatment is observed in 12% to 27%, making surgical management challenging [2, 6, 7]. Intralesional curettage with adjuvant treatment and defect filling is the standard treatment of GCTB with local tumor control and joint preservation [5, 8, 9]. High-speed burring, bone cement or chemical adjuvants (phenol, alcohol, hydrogen peroxide and liquid nitrogen) are usually used to extend the curettage [2, 4].

Polymethylmethacrylate (PMMA) bone cement is most commonly used to augment and fill the cavity. PMMA provides immediate mechanical support, but the main concern is that the hyperthermic reaction may cause articular cartilage damage, and the stiffness of bone cement may decrease the shock absorber joint function [10, 11]. Degenerative arthritis was reported in up to 26% to 33% of patients after curettage and augmentation [1, 4, 6, 11, 12].

The aim of this study was to answer the following questions:

A) What is the incidence of arthritis following treatment of GCTB around the knee by extended curettage and PMMA filling?
B) Are any factors associated with an increased incidence of arthritis in these patients?

## Material and methods

This was a retrospective study of patients with GCTB around the knee who underwent curettage followed by cementation between 1995 and 2012 with a minimum follow-up of 10 years. We excluded patients with tumors not reaching the subchondral bone, patients who developed recurrence after curettage and cementation and were treated with resection and endoprosthetic replacement, and patients with diagnoses other than GCTB, such as aneurysmal bone cyst, chondroblastoma, enchondroma, or low-grade chondrosarcoma. Patients who initially presented with recurrence and underwent re-curettage and cementation were included in the study.

All methods were performed following the ethical standards as laid down in the Declaration of Helsinki and its later amendments or comparable ethical standards. Written informed consent was obtained from all participants or from a parent and/or legal guardian if participants were under 16. The study was conducted after the approval of the Institutional Review Board (IRB) of our university.

Clinical data and radiological evaluations (plain radiographs, CT, or MRI) were reviewed, and the collected data included age, sex, presentation, tumor location, proximity to subchondral bone and exposure of the articular cartilage, pathological fracture, recurrence, metastasis, complications, re-operation, and follow-up period.

If a tourniquet was used, exsanguination was avoided, and it was kept until cementation was completed. The surgical approach was planned according to the tumor location, with an adequate window for wide exposure of the lesion and the removal of pathological tissue using varied sizes of curettes, either straight or curved. Extension of curettage was done with a high-speed burr, with great care taken during its use towards the articular surface, where the burr was used tangentially to avoid penetration of the articular cartilage. The bone cavity was washed out with jet saline throughout the procedure to help dislodge any pathological tissue. The number of cement packs prepared depended on the size of the lesion. Autologous iliac crest bone graft was considered after curettage if there was any breaching of the articular cartilage or if the cortical defect through the planned window was large enough to allow intraarticular leakage of cement. Sometimes additional fixation was performed in cases of pathological fracture or extensive lesions that required more stability. All patients were diagnosed with GCTB both through biopsy and on examination of the final pathological specimen.

Postoperative rehabilitation included muscle strengthening, knee mobilization and immediate weight-bearing as tolerated. In patients with pathological fractures, partial weight-bearing was allowed, which increased to full weight-bearing as the fracture healed.

In the follow-up visits, a clinical examination and conventional radiography were done to rule out any recurrence and to assess function. MRI was done if there was any suspected recurrence. The patients were seen every 3 months in the first postoperative year, then every 6 months for the next 4 years, and yearly thereafter. Any recurrence or complications was recorded.

**Fig. 1:**
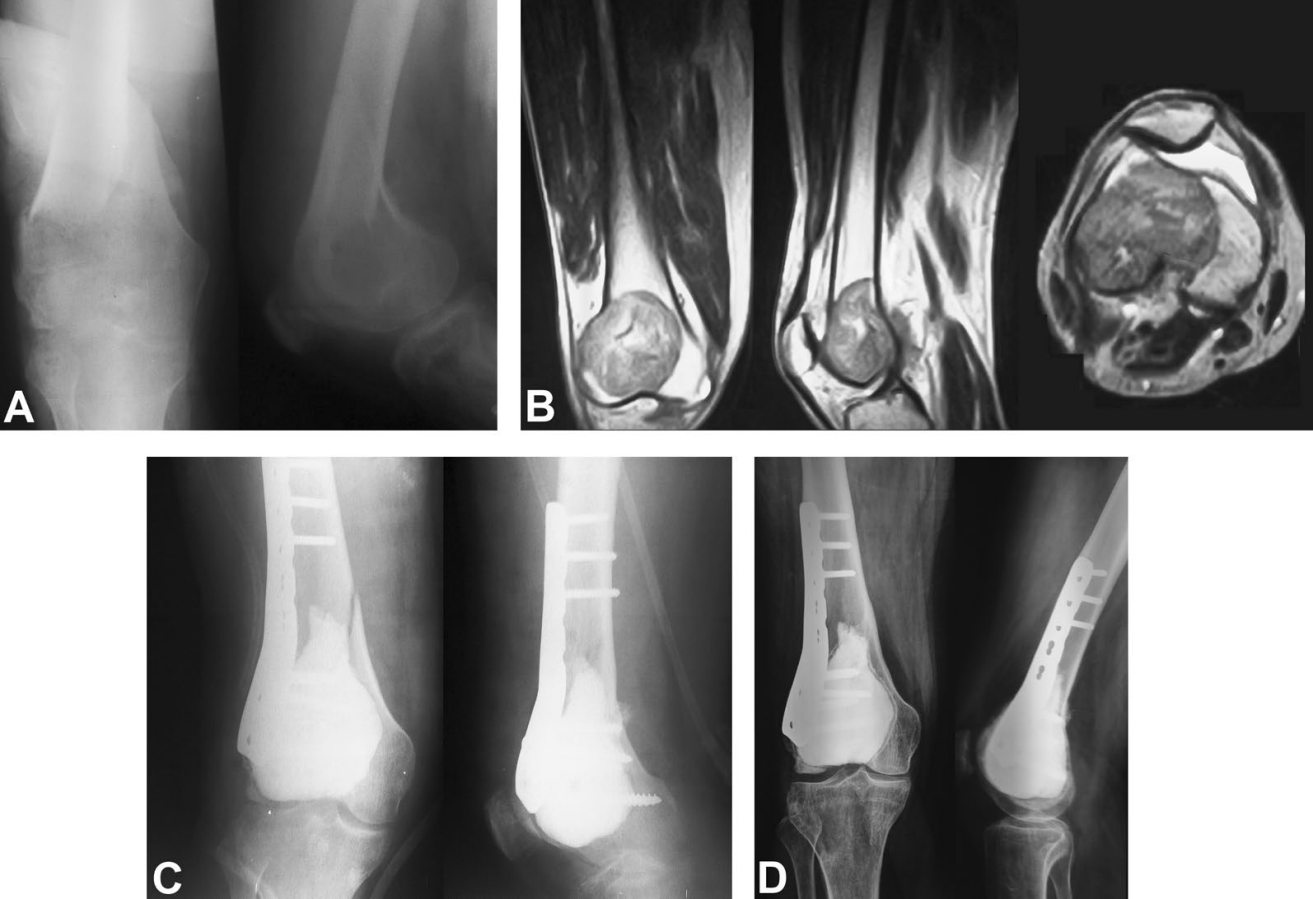
A 29-year-old male with giant cell tumor and pathologic fracture of the distal femur treated with curettage and cementation with distal femur plate fixation. **A** Preoperative AP and lateral X-rays. **B** Preoperative coronal, sagittal, and axial MRI cuts. **C** Immediate postoperative AP and lateral X-rays. **D** Ten-year follow-up AP and lateral X-rays with KL grade 1

**Fig. 2:**
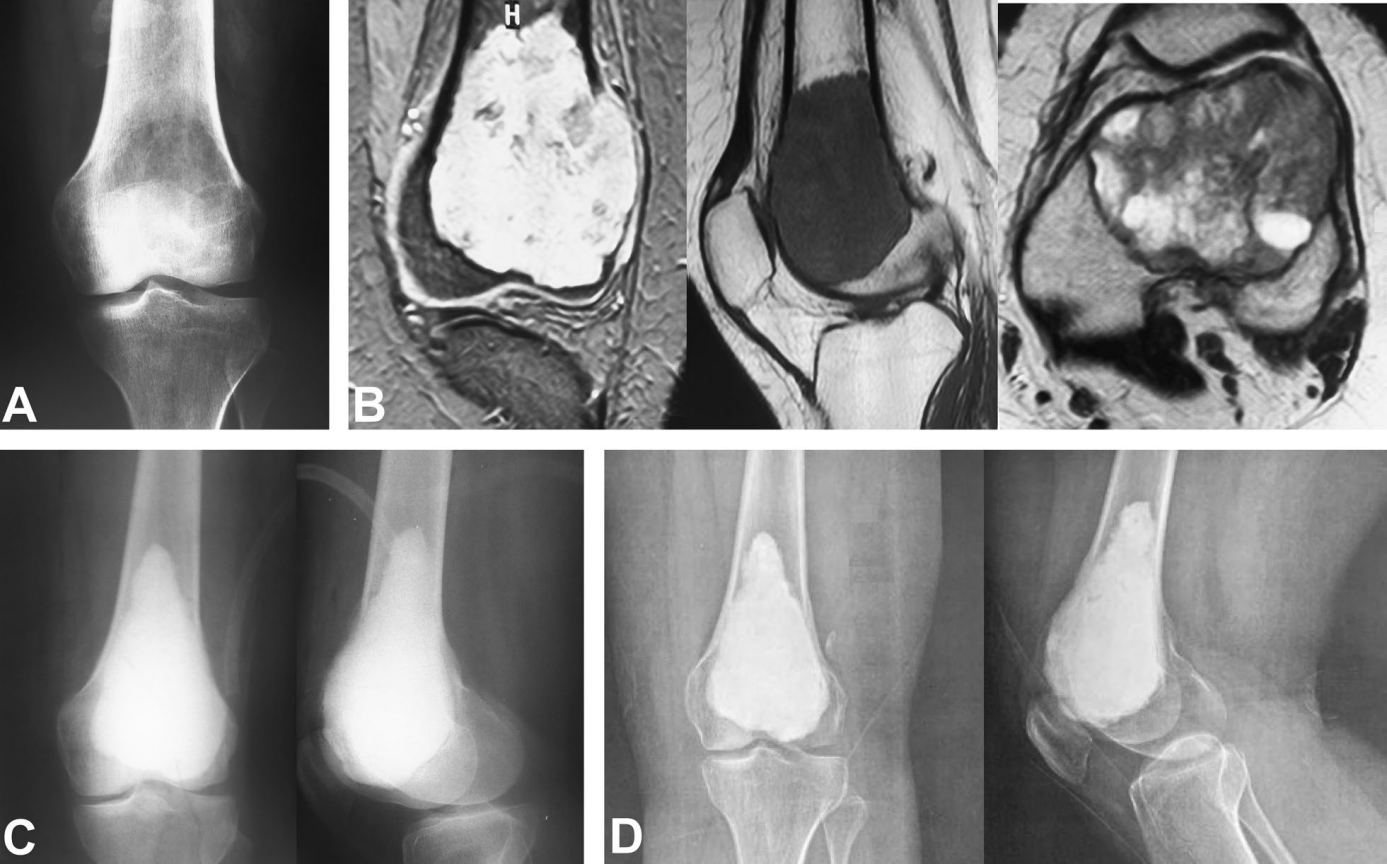
A 25-year-old female with giant cell tumor of the distal femur treated with curettage and cementation. **A** Preoperative AP X-ray. **B** Preoperative coronal, sagittal, and axial MRI cuts. **C** Immediate postoperative AP and lateral X-rays. **D** Ten-year follow-up AP and lateral X-rays with KL grade 1

**Fig. 3:**
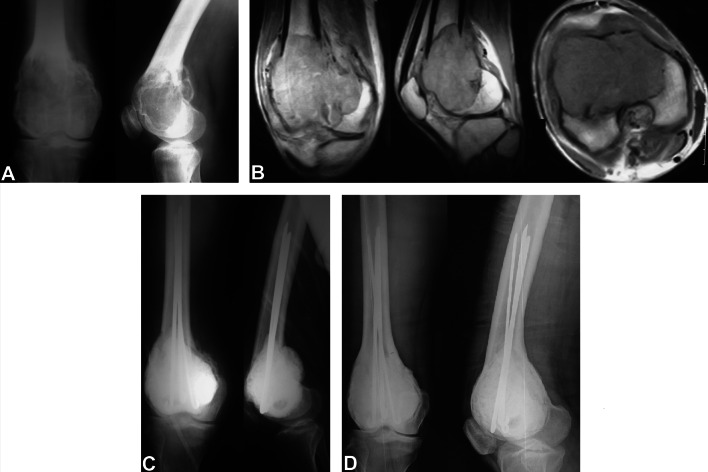
A 28-year-old male with giant cell tumor and pathologic fracture of the distal femur treated with curettage and cementation with fixation using rush pins. **A** Preoperative AP and lateral X-rays. **B** Preoperative coronal, sagittal, and axial MRI cuts. **C** Immediate postoperative AP and lateral X-rays. **D** Ten-year follow-up AP and lateral X-rays with KL grade 3 arthritic changes

The functional outcomes were evaluated using the Musculoskeletal Tumor Society (MSTS) score [13]. Patients were diagnosed with arthritis clinically and radiologically. Arthritis was classified using the Kellgren–Lawrence (KL) classification system of osteoarthritis [14], Figs. 1, 2, and 3.

### Statistical analysis

IBM SPSS version 26.0 (IBM Corp, Armonk, NY) was used for statistical analysis. Quantitative data were expressed as mean ± SD, while qualitative data were expressed as frequency and percentage. The mean MSTS scores were compared between groups using Student’s *t*-test. *P* values of less than 0.05 were considered significant.

## Results

This study included 119 patients, 54 (45.4%) males and 65 (54.6%) females, with a mean age at presentation of 29.4 ± 9.2 (range, 15–56) years. Patients presented with pain (*n* = 89, 74%) or pain and swelling (*n* = 30, 26%). Pathological fractures were encountered in 35 (29.4%) patients.

Regarding tumor location, 70 (58.8%) lesions were located in the distal femur and 49 (41.2%) in the proximal tibia. All tumors reached the subchondral bone, and 92 (77%) tumors were in direct contact with the articular cartilage. Patients presented at our institute with de novo lesions (*n* = 84, 70.6%) or recurrent lesions (*n* = 35, 29.4%); see Table 1.

**Table 1:** Baseline and demographic data for the included patients

Characteristics	Study patients (*n* = 119)
Age, years (mean ± SD)	29.4 ± 9.2
Gender (*n*, %)	
Males	54 (45.4%)
Females	65 (54.6%)
Presentation (*n*, %)	
Pain	89 (74%)
Pain and swelling	30 (26%)
Pathological fractures (n, %)	
Yes	35 (29.4%)
No	84 (70.6%)
Tumor location (n, %)	
Distal femur	70 (58.8%)
Proximal tibia	49 (41.2%)
Recurrent at presentation (n, %)	
De novo	84 (70.6%)
Recurrent	35 (29.4%)

No patients had arthritis in the preoperative plain X-rays. Patients did not receive denosumab before surgery. A high-speed burr was used in all patients except 4 patients who were operated on before the adoption of the extended curettage technique. Hydrogen peroxide was used in 28 (23.5%) patients.

The mean number of bone cement packs (40 gm) used was 2.5 ± 0.95 (range, 1–5). An iliac crest bone graft towards the articular surface was used in 5 (4.2%) patients. Additional internal fixation procedures were done in 16 (13.4%) patients using Steinmann pins (*n* = 8) or plate osteosynthesis (*n* = 8). The mean operative time was 1.69 ± 0.55 (range, 1–3) h.

The mean follow-up period was 13.2 ± 3.16 (range, 10–22) years.

The mean MSTS score was 28.5 ± 1.9 (range, 20–30). Patients who developed arthritis showed a lower mean MSTS score at the final follow-up than those without arthritis: 27.3 ± 2.6 and 28.8 ± 1.4, respectively; *P* = 0.009.

Regarding the range of motion, 109 (91.6%) patients achieved the full range of knee flexion and extension, while 10 (8.4%) patients had a reduced range of motion of 5–110° (*n* = 1), 0–100° (*n* = 2), 0–90° (*n* = 2), 20–100° (*n* = 2), 0–80° (*n* = 1), 20–90° (*n* = 1), and 0–60° (*n* = 1). Four patients had flexion deformities.

Out of all the patients, 15 (12.6%) patients had occasional knee pain on prolonged walking, exertion, going upstairs or downstairs, and deep flexion, with no arthritic changes on radiographic evaluation. Those patients were classified as KL grade 0 and responded well to physiotherapy, knee exercises, and weight reduction for overweight patients. The pain was attributed to mild patellofemoral arthritis or chondromalacia patellae.

Overall, 25 (21%) patients developed variable degrees of arthritis. Out of those 25 patients, 7 (28%) patients had KL grade 1, 11 (44%) KL grade 2, 4 (16%) KL grade 3, and 3 (12%) KL grade 4. All patients had a satisfactory response to conservative measures, including weight loss, pain medications, and physiotherapy, except 4 patients (1 patient with grade 3 who had valgus deformity and 3 patients with grade 4). Those 4 patients were offered total knee replacement but only one with grade 4 accepted; the other 3 patients refused and preferred to continue with conservative measures.

Ten (8.4%) patients had a progression of arthritis with a progression of the KL grade during the follow-up period, while the remaining 15 maintained the same KL grade during the follow-up period.

The mean age at presentation of patients who developed secondary osteoarthritis was similar to that of patients who did not develop osteoarthritis: 29.3 ± 9.3 and 29.4 ± 9.3 years, respectively;* P* = 0.958. Moreover, other factors, including gender, presence of pathological fracture at presentation, whether the tumor was de novo or recurrent at presentation, and the tumor location, were not associated with the incidence of osteoarthritis; see Table 2.

**Table 2:** Factors affecting the incidence of secondary osteoarthritis

Characteristics	Osteoarthritis (*n* = 25)	No osteoarthritis (*n* = 94)	*P* value
Age, years (mean ± SD)	29.3 ± 9.3	29.4 ± 9.3	0.958
Gender			0.543
Males (*n* = 54)	10	44	
Females (*n* = 65)	15	50	
Pathological fracture at presentation			0.504
Yes (*n* = 35)	6	29	
No (*n* = 84)	19	65	
Tumor location			0.554
Distal femur (*n* = 70)	16	54	
Proximal tibia (*n* = 49)	9	40	
Recurrent at presentation			0.862
De novo (*n* = 84)	18	66	
Recurrent (*n* = 35)	7	28

Other reported complications were acute deep venous thrombosis (*n* = 1) and deep infections with sinus (*n* = 2). The two patients with deep infection refused to have any surgical debridement, and they had good response to antibiotics. Two other patients were subjected to a fall and had a fracture proximal to the inserted cement. One was treated with ORIF, and the other was treated conservatively. No patients had instability.

Local recurrences occurred in 19 (15.9%) patients. Two of them had soft-tissue recurrences. All the recurrences appeared during the first 2 years of follow-up. These patients were treated by re-curettage and cementation. Patients with a soft-tissue recurrence were treated by excision. None of these patients developed any recurrence after that.

## Discussion

GCTB is usually located in the epiphyseal-metaphyseal area and often destroys subchondral bone. Curettage followed by cementation is the standard surgical treatment option for GCTB [7, 9, 12, 15, 16].

In this study, we evaluated long-term functional and radiological results, including the incidence and progression of osteoarthritis following curettage and cementation of GCTB around the knee. After an average follow-up of 13.2 years, the overall incidence of osteoarthritis was 21%. Additionally, 40% of those patients had a progressive course based on the KL grading system of osteoarthritis. Secondary arthritic changes were reported in the literature to occur in 26 to 33% of patients who had curettage and augmentation using PMMA or calcium phosphate cement [1, 4, 6, 11, 12]. With an average follow-up of 32.9 ± 7.1 months, Wu et al. [17] studied 27 patients with GCTB around the knee who had subchondral bone grafting and bone cement reconstruction and reported 2 (7.4%) patients with arthritic changes. Wechsler et al. [4] evaluated the knee joint degeneration following curettage and cementation of GCTB in 14 patients and reported a progression of KL grade in 3 (21%) patients with an average follow-up of 54.6 months.

The mean overall MSTS score in our study was 28.5 ± 1.9, and patients with arthritis had significantly lower scores (27.3 ± 2.6) than those who were not arthritic (28.8 ± 1.4). Non-arthritic patients and those with grade one or two arthritis could perform the activities of daily living. All patients with arthritis were managed conservatively, and only 1 patient was managed by TKR.

Lee et al. [18] had six joint replacements in 40 patients with GCTB treated with intralesional curettage, and reported that arthroplasty was performed as a secondary procedure in patients with GCTB at a relatively infrequent rate and more often for cases of recurrent disease than for osteoarthritis. Wechsler et al. [4] reported that 1 out of 14 patients had a joint replacement.

The follow-up period in the current study was long enough to evaluate the occurrence and progression of osteoarthritis. Several studies reported no arthritic changes after curettage and cementation of GCTB [16, 19, 20]. Benevenia et al. [21] compared the postoperative complications in patients with GCT of long bone epiphysis treated with curettage and by filling the cavity with PMMA only (*n* = 22 patients) versus bone graft with or without supplemental PMMA (*n* = 21 patients). The overall nononcologic complications were significantly lower in the bone graft group. The rates of osteoarthritis were higher in the PMMA-only group (*n* = 7) compared to the bone graft group (*n* = 1) [21]. Gaston et al. [22] compared curettage alone versus curettage and PMMA cementation in the management of GCTB and found that an increased incidence of osteoarthritis and a subsequent need for joint replacement were associated with using PMMA.

Szalay et al. [23] compared curettage with the use of bone graft versus cementation in treating GCTBs and reported arthritis rates of 19.4% and 15.9% in patients with cementing and bone grafting, respectively, at the 50-month follow-up.

In our study, great care was taken not to penetrate or break articular cartilage, especially while using curettes and a high-speed burr. On some occasions, iliac crest bone graft was used towards articular cartilage and subchondral bone. The use of bone graft toward the subchondral bone and cartilage, together with PMMA, has been reported to reduce the risk of osteoarthritis or mechanical failure [17, 24, 25]. At a mean of 33 months, Teng et al. [24] found that combined grafting and cementation reduce the risk of mechanical failure in the knee due to the thin subchondral bone layer, especially in the femur. Wechsler et al. [4] tried to minimize the adverse biomechanical effect of bone cement on the surrounding articular cartilage through removal followed by autografting of the defect, but there was no statistically significant difference compared to those retained in the primary cementation.

Being a tertiary center, we receive a large number of patients with previous interventions, which is why 29.4% of patients were diagnosed as recurrent cases. However, after surgical intervention, the recurrence rate was 15.9%. The rate of recurrence in the final cohort may be low due to the exclusion of cases that had prosthetic reconstruction for recurrence after cementation and due to being located around the knee, as compared to the proximal femur and distal radius, which have a high recurrence rate [15]. In a review study performed by Errani et al. [15], 26 studies were included, and all were after 2000, with an overall local recurrence rate of 0 to 65%.

Some authors found that using PMMA after curettage resulted in a reduction of the local recurrence rate, permitting immediate weight-bearing and more efficient detection of local recurrence [4, 22, 26, 27]. Others, however, found no significant effect of filling the cavity with PMMA on local recurrence [21, 28]. Adjuvant treatment or advanced operative techniques resulted in a lower recurrence rate [29, 30].

This study is not without limitations. This was a retrospective study, but it included a large study population in a single institution. Our control group for the occurrence of osteoarthritis was the contralateral healthy side, but it was difficult to find a control group receiving PMMA in direct proximity to the articular cartilage for an actual prediction of true degenerative potential, as there are no other disorders that require this technique except bone tumors reaching the subchondral bone.

## Conclusion

Giant cell tumors of bone around the knee could be safely and predictably treated with curettage and by filling the cavity with cement. Knee arthritis is a possible complication; however, mild grades of arthritis are predicted in most patients. A small percentage of patients could get advanced grades and could be offered knee replacement. No correlation was found between the incidence of arthritis and age, gender, presence of pathological fracture, tumor location, or recurrent tumor at presentation.

## Data Availability

The dataset analyzed in this study is available from the corresponding author on request.
